# Supplementary figures and images for: Reduced STMN2 and pathogenic TDP-43, two hallmarks of ALS, synergize to accelerate motor decline in mice
Source: bioRxiv. 2024 Mar 20:2024.03.19.585052. Preprint. [Version 1] doi: 10.1101/2024.03.19.585052 (PMC10983882; doi:10.1101/2024.03.19.585052)

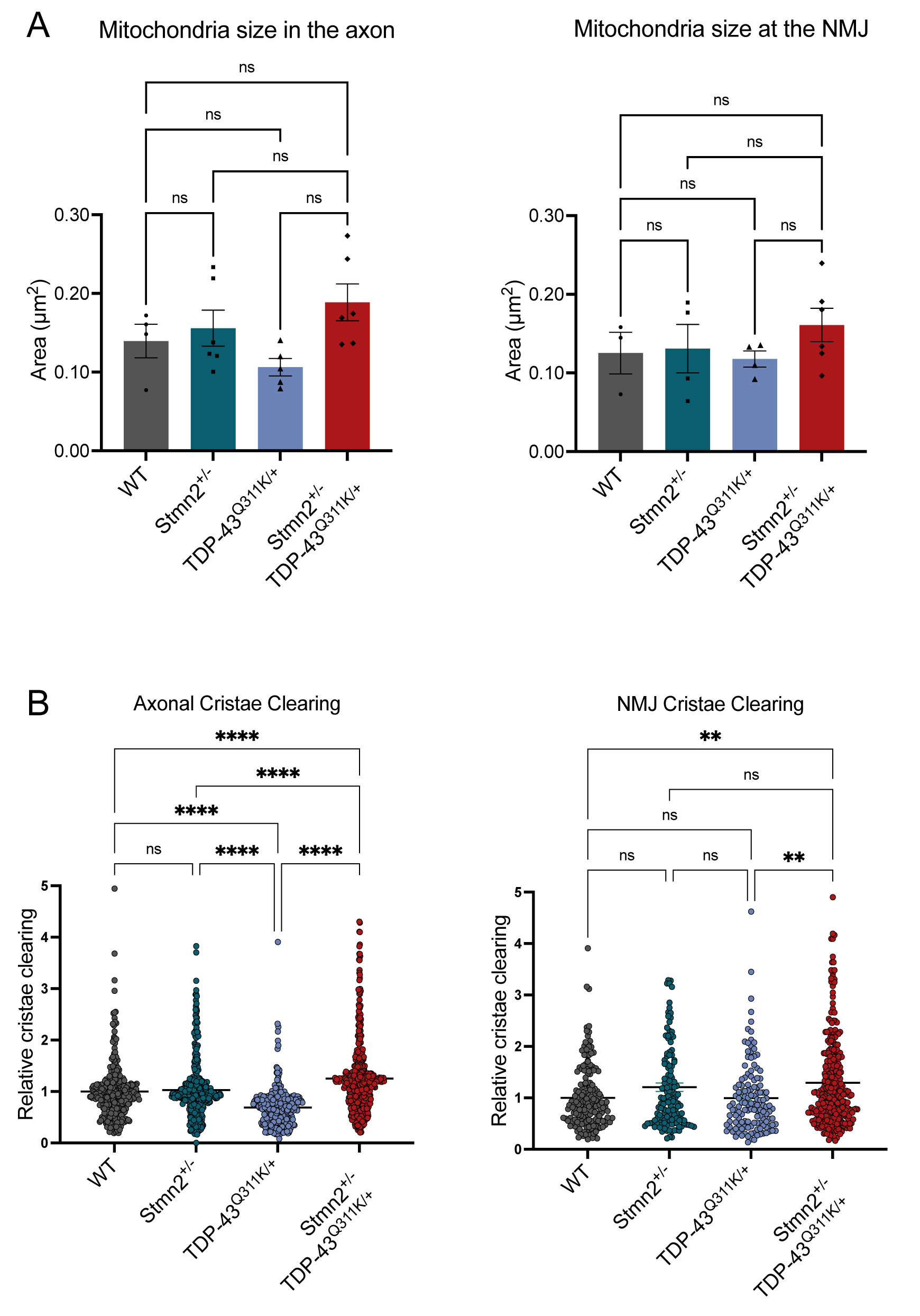

Supplement: Supplement 1 — A) Average mitochondrial area in distal axons and NMJs in lumbrical muscles of WT, TDP-43Q331K, Stmn2+/−, and TDP-43Q331K;Stmn2+/− mice. Each point represents the average area of multiple mitochondria from a single animal. B) Mitochondrial cristae density in distal axons and NMJs in lumbrical muscles of WT, TDP-43Q331K, Stmn2+/−, and TDP-43Q331K;Stmn2+/− mice calculated using the mean grey value of each mitochondrion normalized to the average grey value for all wildtype mitochondria. A larger value indicates a greater area of the mitochondrion free of dense cristae. Each point represents one mitochondrion. [file media-1.tif]
